# Hybrid pedicle screw and modified cortical bone trajectory technique in transforaminal lumbar interbody fusion at L4-L5 segment: finite element analysis

**DOI:** 10.1186/s12891-023-06385-y

**Published:** 2023-04-13

**Authors:** Alafate Kahaer, Rui Zhang, Yixi Wang, Haopeng Luan, Abulikemu Maimaiti, Dongshan Liu, Wenjie Shi, Tao Zhang, Hailong Guo, Paerhati Rexiti

**Affiliations:** 1 Department of Spine Surgery, The First Affiliated Hospital of Xinjiang Medical University, 137 Liyushan Road, Urumqi, China; 2 First Clinical Medical Institution, Xinjiang Medical University, Urumqi, China; 3 Digital Orthopaedic Center of Xinjiang Medical University, Urumqi, China

**Keywords:** Pedicle screw, Modified cortical bone trajectory, Transforaminal lumbar interbody fusion, Finite element analysis, Hybrid fixation

## Abstract

**Background:**

Investigate the biomechanical properties of the hybrid fixation technique with bilateral pedicle screw (BPS) and bilateral modified cortical bone trajectory screw (BMCS) in L4-L5 transforaminal lumbar interbody fusion (TLIF).

**Methods:**

Three finite element (FE) models of the L1-S1 lumbar spine were established according to the three human cadaveric lumbar specimens. BPS-BMCS (BPS at L4 and BMCS at L5), BMCS-BPS (BMCS at L4 and BPS at L5), BPS-BPS (BPS at L4 and L5), and BMCS-BMCS (BMCS at L4 and L5) were implanted into the L4-L5 segment of each FE model. The range of motion (ROM) of the L4-L5 segment, von Mises stress of the fixation, intervertebral cage, and rod were compared under a 400-N compressive load with 7.5 Nm moments in flexion, extension, bending, and rotation.

**Results:**

BPS-BMCS technique has the lowest ROM in extension and rotation, and BMCS-BMCS technique has the lowest ROM in flexion and lateral bending. The BMCS-BMCS technique showed maximal cage stress in flexion and lateral bending, and the BPS-BPS technique in extension and rotation. Compared to the BPS-BPS and BMCS-BMCS technique, BPS-BMCS technique presented a lower risk of screw breakage and BMCS-BPS technique presented a lower risk of rod breakage.

**Conclusion:**

The results of this study support that the use of the BPS-BMCS and BMCS-BPS techniques in TLIF surgery for offering the superior stability and a lower risk of cage subsidence and instrument-related complication.

## Introduction

The number of fusion surgery performed for the spinal degenerative diseases is gradually rising [1]. Transforaminal lumbar interbody fusion (TLIF) has become a commonly used surgical approach, since Harms and Rolinger introduced it as an alternate technique in 1982 [2]. Previous studies have shown TLIF to be superior compared to posterior lumbar interbody fusion (PLIF), including lower surgery related complications as infections, nerve root damage, dural tears, instrument-related problems [3], lower duration of surgery, estimated blood loss, and financial burden, while the clinical outcome seems to be similar [3, 4].

**Fig. 1:**
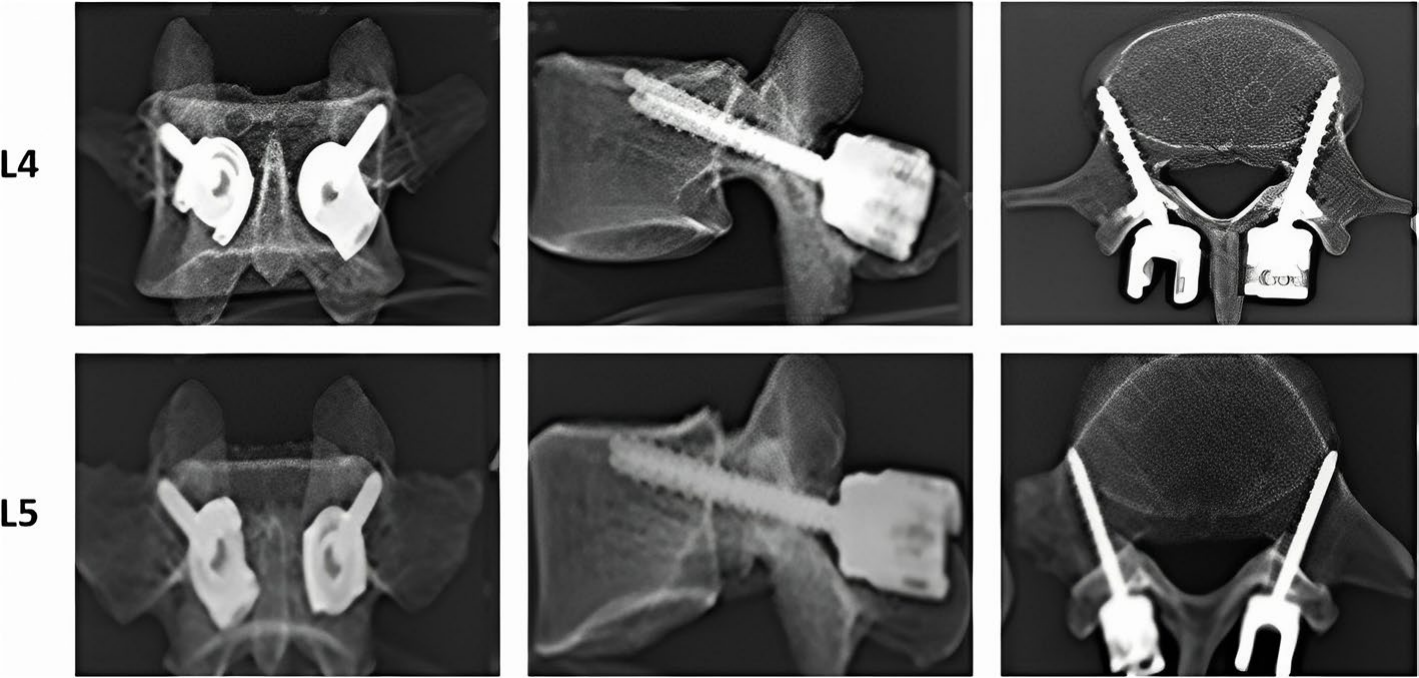
Schematic diagram of the MCBT technique at L4 and L5 [6]. MCBT technique reaches the medial wall of the pedicle and the lateral margin of the superior endplate

Traditional trajectory (TT) screw has been the gold standard in spine surgery [5]. It was widely used in TLIF and PLIF procedures. However, in recent years, due to complications such as decreased stability of the fixation unit caused by screw loosening, adjacent segment degeneration (ASD) caused by the violation to the facet joints, fixation in some patients cannot obtain a superior therapeutic effect and require revision, which is more common in elderly patients [6]. The main reason for screw loosening is that pedicle screws are mainly fixed by cancelous bone, and it is often compromised in patients with osteoporosis. Cortical bone trajectory (CBT) technique, proposed by Santoni et al. [6], could compensate for the shortcomings of pedicle screw, but it failed to utilize cortical bone throughout its trajectory. To compensate for the shortcomings of CBT and TT, we previously proposed a modified cortical bone trajectory (MCBT) technique [7–9], which reaches the cortical bone located at the medial wall of the pedicle and the lateral margin of the superior endplate by moving the insertion point medially, increasing the medio-lateral angle, and reducing the cranio-caudal angle compared to the CBT technique (Fig. 1). If the screw entry point did not move medially, damage of the screw entry point and splitting of the lateral wall of the pedicle would be occured while increasing the medio-lateral angle. In addition, we proposed the hybrid fixation technique with MCBT and TT [10], and demonstrated its superior biomechanical properties in the L4-L5 segment without fusion [10]. However, the hybrid technique in the TLIF model remains unclear. In this study, the biomechanical properties of the hybrid BPS-BMCS and BMCS-BPS techniques were investigated in the L4-L5 TLIF model using finite element (FE) analysis.

## Materials and methods

### Development of an intact L1-S1 finite element model

**Fig. 2:**
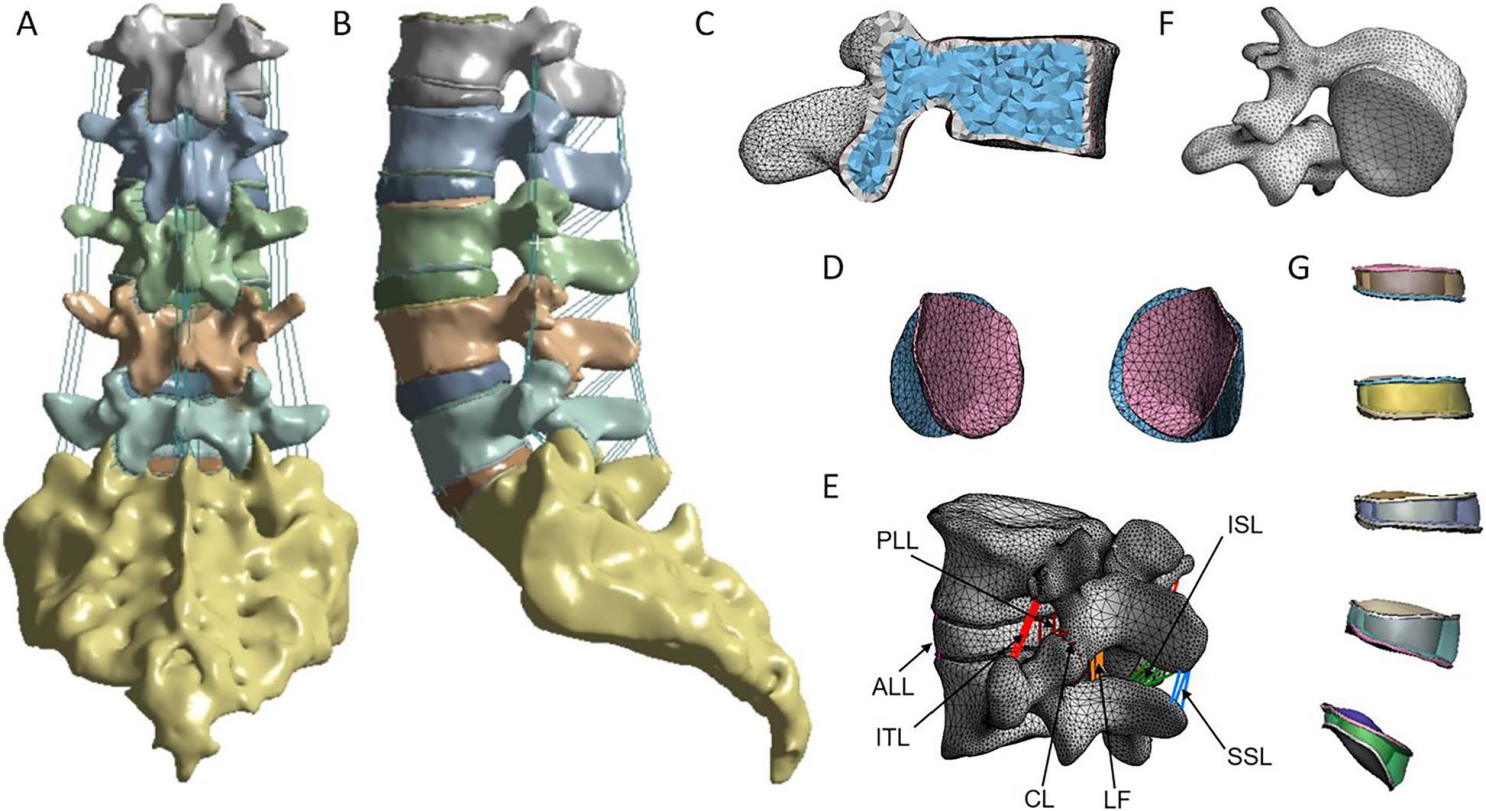
The FE model of the intact L1-S1 lumbar spine. **A** Back view, **B** Sagittal view, **C** Regional thickness of the cortical bone, **D** Facet cartilage, **E** Ligaments, **F** Vertebral endplate, **G** Intervertebral discs

Three specimens were selected from the Anatomy Teaching-Research Office of Xinjiang Medical University. The mean age of them was 71, ranging from 64 to 77 years. Bone mineral density (BMD) indicating osteoporosis (BMD T < -0.25 SD), with the exclusion of previous lumbar spine surgery, infection, and tumor. High-resolution computed tomography (CT) data (AQUIRRON 16, PHILIPS, Netherlands) of L1-S1were collected and stored in DICOM format. Import CT data of L1-S1 into Mimics 17.0 (Materialize, Leuven, Belgium) software, and define the orientations. Select the “bone” threshold selection tool, select the appropriate range of vertebral body, fill in the vacancy, remove the redundant area with the brush tool, process the CT layer by layer. Afterward, the 3D models were saved as an STL format and imported into 3-Matic 11.0 software (Materialise, Leuven, Belgium) for further processing. The models were re-meshed to reduce the risk of distortion during the subsequent smoothing process, and the details of the model were refined. The intact L1-S1 FE models were composed of 5 lumbar vertebrae with the sacrum (Fig. 2 A, B), facet joint cartilages at each segment (Fig. 2 D), 7 ligaments (Fig. 2 E), 5 intervertebral discs with the cranial and caudal endplates (Fig. 2 F, G). The thickness of cortical bone (Fig. 2 C) and endplate were defined as 0.5-1 mm [11] and 1 mm [12] respectively. The nucleus pulposus was modeled as an incompressible fluid-filled cavity with low stiffness occupying 44% of the disc volume [13], The contact between the facet cartilages was defined as “soft frictionless contact” with an initial gap of 0.5 mm [11]. The anterior longitudinal ligament (ALL), posterior longitudinal ligament (PLL), intertransverse ligament (ITL), ligamentum flavum (LF), capsular ligament (CL), interspinous ligament (ISL), and supraspinous ligament (SSL) were represented and assigned nonlinear material properties (Fig. 2 E). At last, the meshed models were processed using ANSYS Workbench 19.1 (ANSYS, Inc., Canonsburg, PA, USA) to set the material properties [14–18] (Table 1).

**Table 1:** Material properties in current study [14–18]

Materials	Young’s Modulus (Mpa)	Poisson’s Ratio	Density (g/cm3)	Cross Sectional Area (mm2)	Radius(mm)
Cortical bone	12,000	0.3	1.91		
Cancellous bone	100	0.2	1.87		
Cartilaginous endplate	23.8	0.4	1.0003		
Facet cartilage	24	0.4			
Annulus fibrosis	4.2	0.45			
Nucleus pulposus	1	0.4999			
ALL	7.8(< 12.0%) 20.0(> 12.0%)		1.00E-06	63.7	4.5029
PLL	10.0(< 11.0%) 20.0(> 11.0%)		1.00E-06	20	2.5231
CL	7.5(< 25.0%) 32.9(> 25.0%)		1.00E-06	30	3.0902
LF	15.0(< 6.2%) 19.5(> 6.2%)		1.00E-06	40	3.5682
ISL	10.0(< 14.0%) 11.6(> 14.0%)		1.00E-06	40	3.5682
SSL	8.0(< 20.0%) 15(> 20.0%)		1.00E-06	30	3.0902
ITL	10.0(< 18.0%) 58.7(> 18.0%)		1.00E-06	1.8	0.7569
Cage(PEEK)	3600	0.25	1.32e − 6		
Screw and Rod(Titanium)	110,000	0.3	4.5e − 6		

### Model validation

The validation of the intact FE models includes two steps. First, the mesh convergence test was performed for 1 of the 3 intact FE models. For this FE model, 3 different mesh resolutions (mesh 1, mesh 2, and mesh 3) were generated consecutively. Among the three mesh resolutions, mesh 3 has the highest number of elements and nodes, and mesh 1 has the least. Ayturk et al. [19] demonstrated that axial rotation is the most sensitive to the mesh resolution of the FE model. The rotation was simulated with a torque of 7.5 Nm and the von Mises stresses of different components were compared, and the meshes were considered converged when the difference between the predictions obtained by two successive mesh resolutions was less than 5%. In this study, the differences between the von Mises stresses of the three meshes were compared by the same method. Second, a 400 N compressive load and 7.5 Nm moments were applied to simulate flexion, extension, lateral bending, and rotation, and the range of motion (ROM) of each segment was compared with those of Yamamoto et al. [20], Shim et al. [21], Huang et al. [22], Lo et al. [23].

### Construction of surgical models

**Fig. 3:**
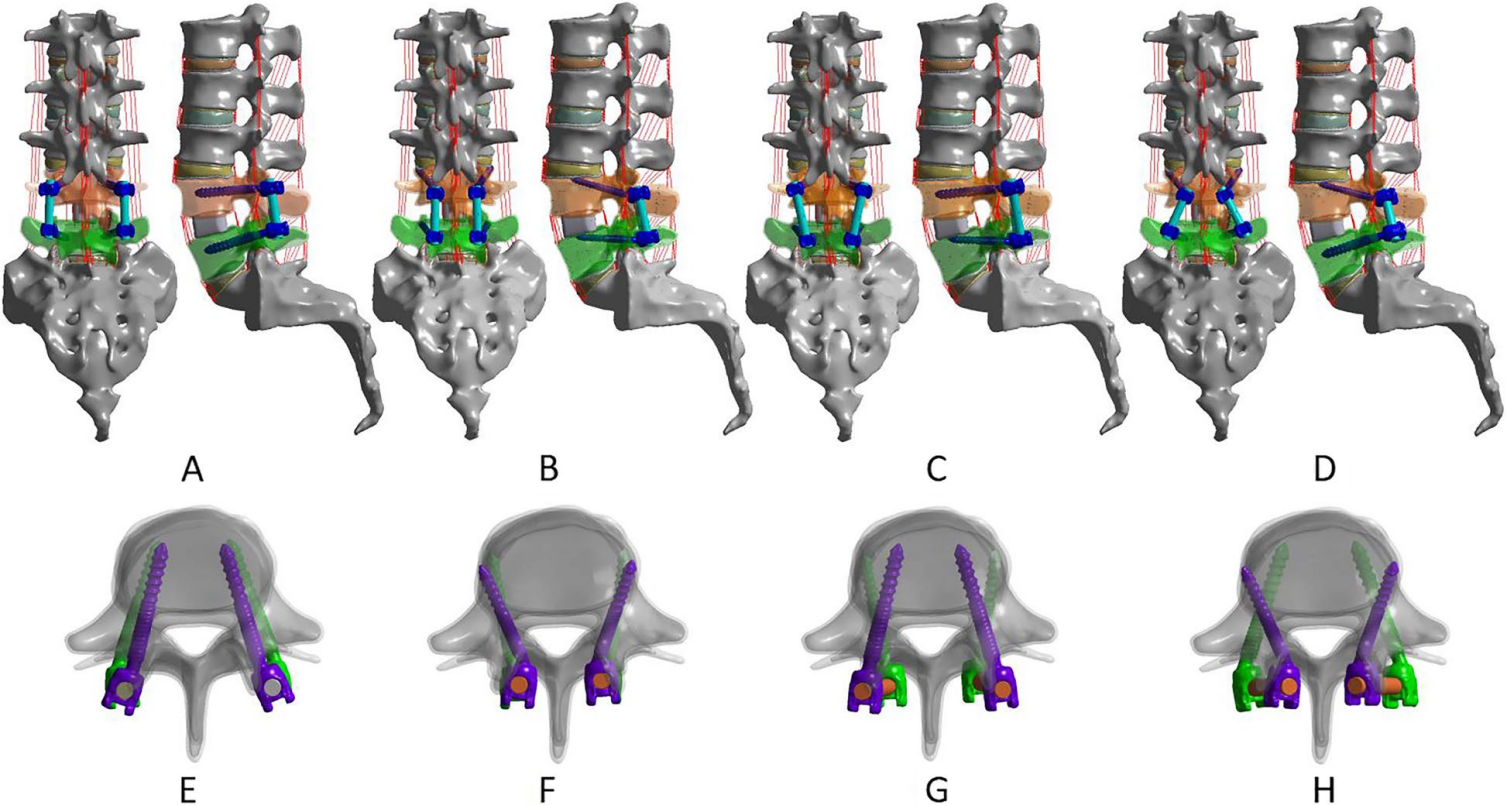
FE models of the L1-S1 lumbar spine with TLIF at the L4-L5 segment with four different fixation techniques. **A** TT screws at L4 and L5 (BPS-BPS). **B** MCBT screws at L4 and L5 (BMCS-BMCS). **C** TT screws at L4 and MCBT screws at L5 (BPS-BMCS). **D** MCBT screws at L4 and TT screws at L5 (BMCS-BPS), and **E–H** were the axial and sagittal views of each technique of **A-D**

TLIF procedures were performed by removing the facet joint and part of the lamina at one side. The CBT screw is moving 25° cranially in the sagittal plane and 10° laterally in the axial plane [24] and the insertion point is located at the lateral aspect of the pars interarticularis projecting in the 7 o’clock orientation in the right pedicle and the 5 o’clock orientation in the left pedicle, when using the clock face for orientation [25]. As previously shown [7–9], the MCBT reaches the cortical bone located at the medial wall of the pedicle and the lateral margin of the superior endplate by moving the insertion point medially, increasing the medio-lateral angle, and reducing the cranio-caudal angle compared to the CBT technique (Fig. 1). The TT screw with a diameter of 6.0 mm and length of 45 mm, the MCBT screw with a diameter of 5.0 mm and length of 40 mm. Four different fixation groups were reconstructed: (1) BPS-BPS group, TT at L4 and L5 (Fig. 3 A, E); (2) BMCS-BMCS group, MCBT at L4 and L5 (Fig. 3 B, F); (3) BPS-BMCS group, TT at L4, and MCBT at L5 (Fig. 3 C, G); and (4) BMCS-BPS group, MCBT at L4, and TT at L5 (Fig. 3 D, H). Four FE models were reconstructed for each specimen. Finally, the reconstructed FE models were imported into ANSYS Workbench 19.1 (ANSYS, Inc., Canonsburg, PA, USA) for further biomechanical analysis.,

### Boundary and loading conditions

**Fig. 4:**
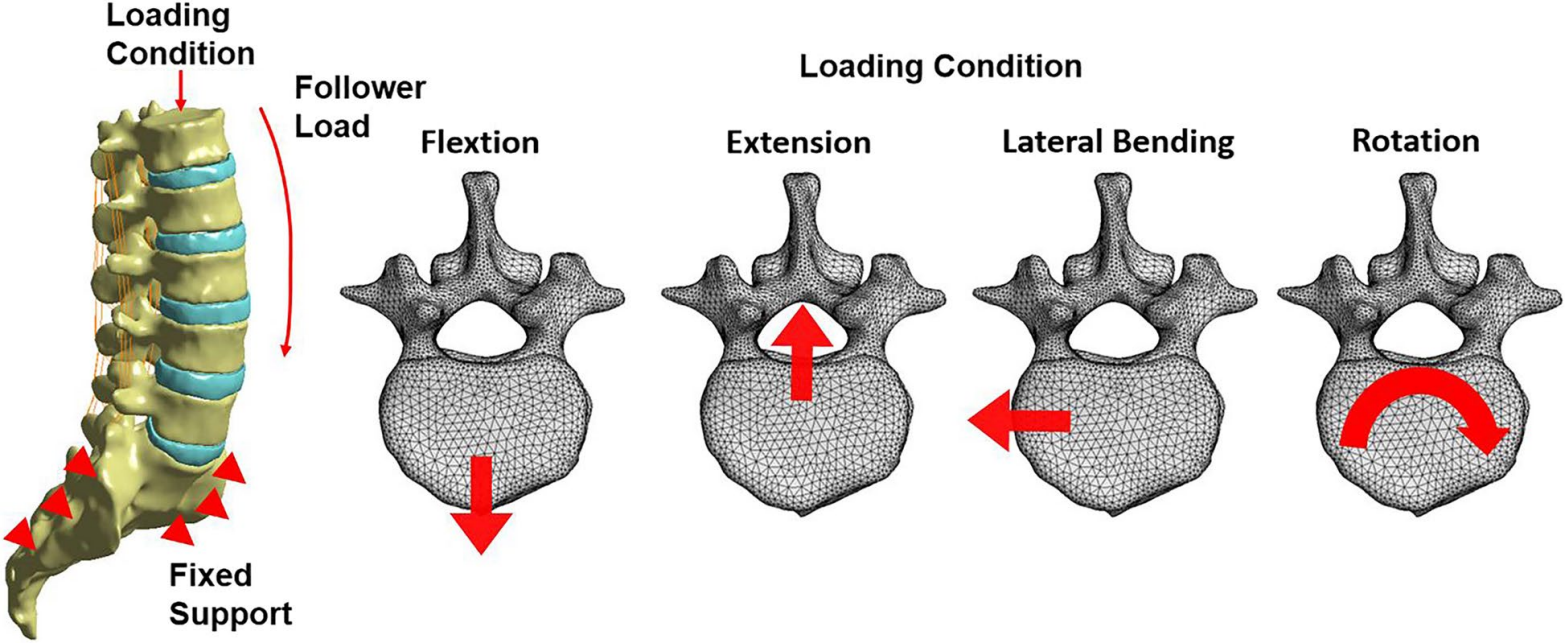
Boundary and Loading conditions

The sacrum was completely fixed and constrained to avoid displacement and rotation. Established a reference point in the center of the upper endplate of L1, and the contact between the reference point and endplate was defined as “contact” constraint. 400 N compressive load and 7.5 Nm torque were applied for simulating flexion, extension, lateral bending, and rotation (Fig. 4). The ROM of the L4-L5 segment, von Mises stress of the screw, intervertebral cage, and rod were recorded and discussed.

### Statistical analysis

SPSS 26.0 software was used for data analysis. The data were expressed as the mean ± standard deviation. One-way analysis of variance (ANOVA) was used for the analysis of differences. Post hoc tests were performed using the LSD method when differences were statistically significant. *P* < 0.05 was considered statistically significant.

## Results

### Model validation

**Fig. 5:**
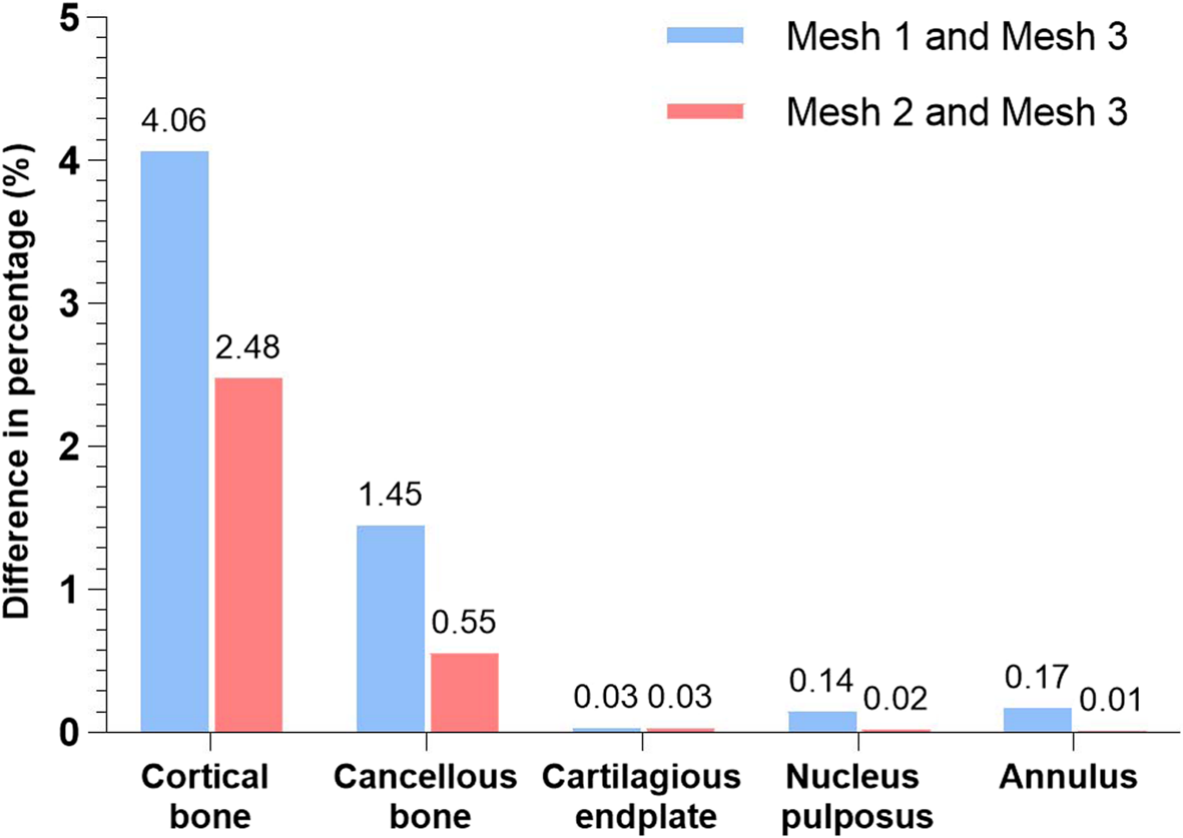
Predicted percentage differences of the von Mises stress between Mesh 1 and Mesh 3 and between Mesh 2 and Mesh 3 in each component

**Fig. 6:**
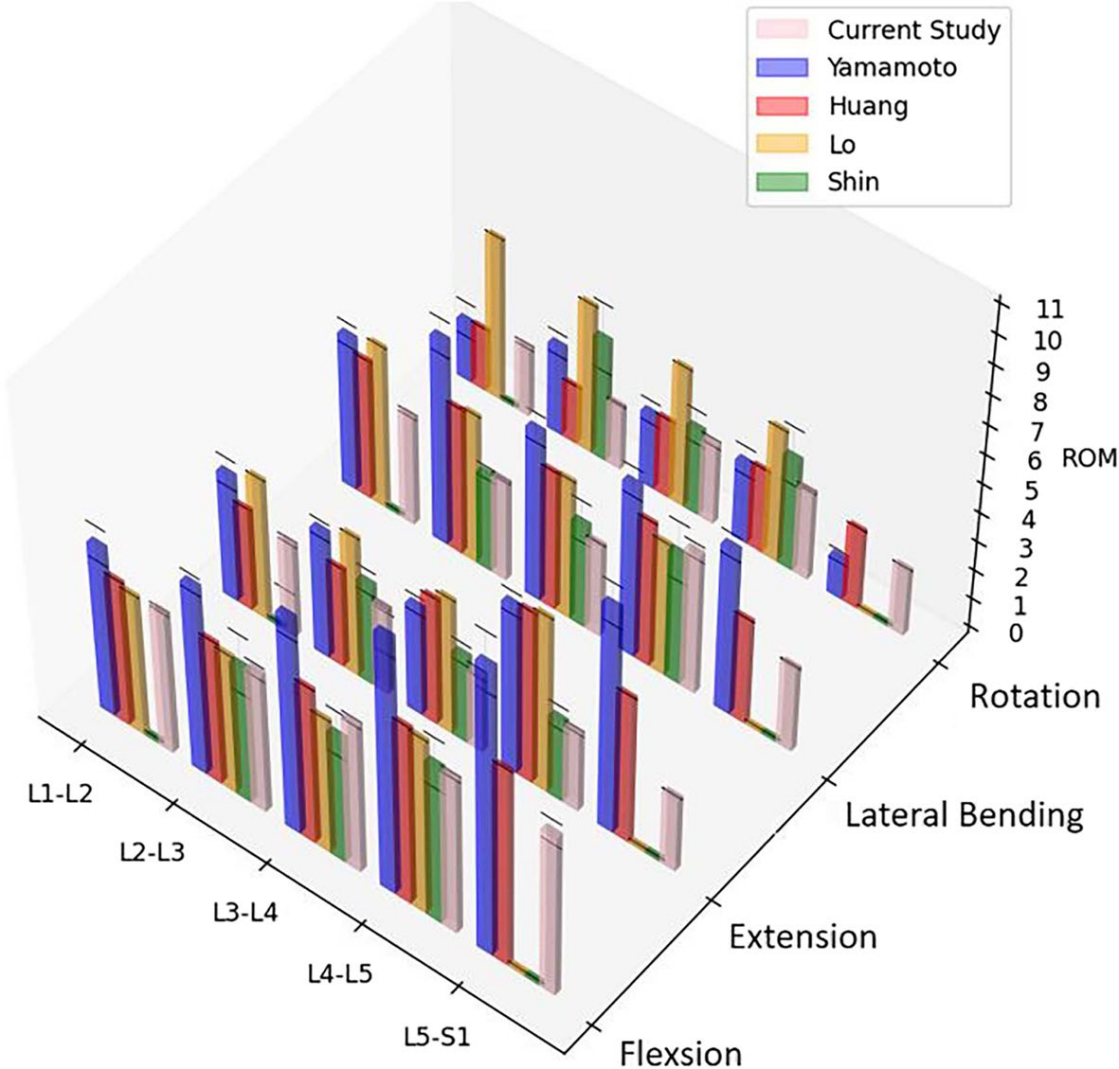
Comparison of ROM of each segment in the current intact FE model with the previous studies

Figure 5 showed the difference of percentage in von Mises stress between the mesh 1 and mesh 3 and between mesh 2 and mesh 3. The greatest difference in predicted von Mises stresses between mesh 1 and mesh 3 was found in the cortical bone (4.06%). The differences in von Mises stress of each component between mesh 2 and mesh 3 was the least. Therefore, mesh 2 was selected. The ROM of the intact model at each segment was similar to the results and variation trends of Yamamoto et al. [20], Shim et al. [21], Huang et al. [22], Lo et al. [23] (Fig. 6), indicating that the intact L1-S1 FE models in this study were successfully constructed and can be used for further biomechanical analysis.

### ROM of the L4-L5 segment

**Fig. 7:**
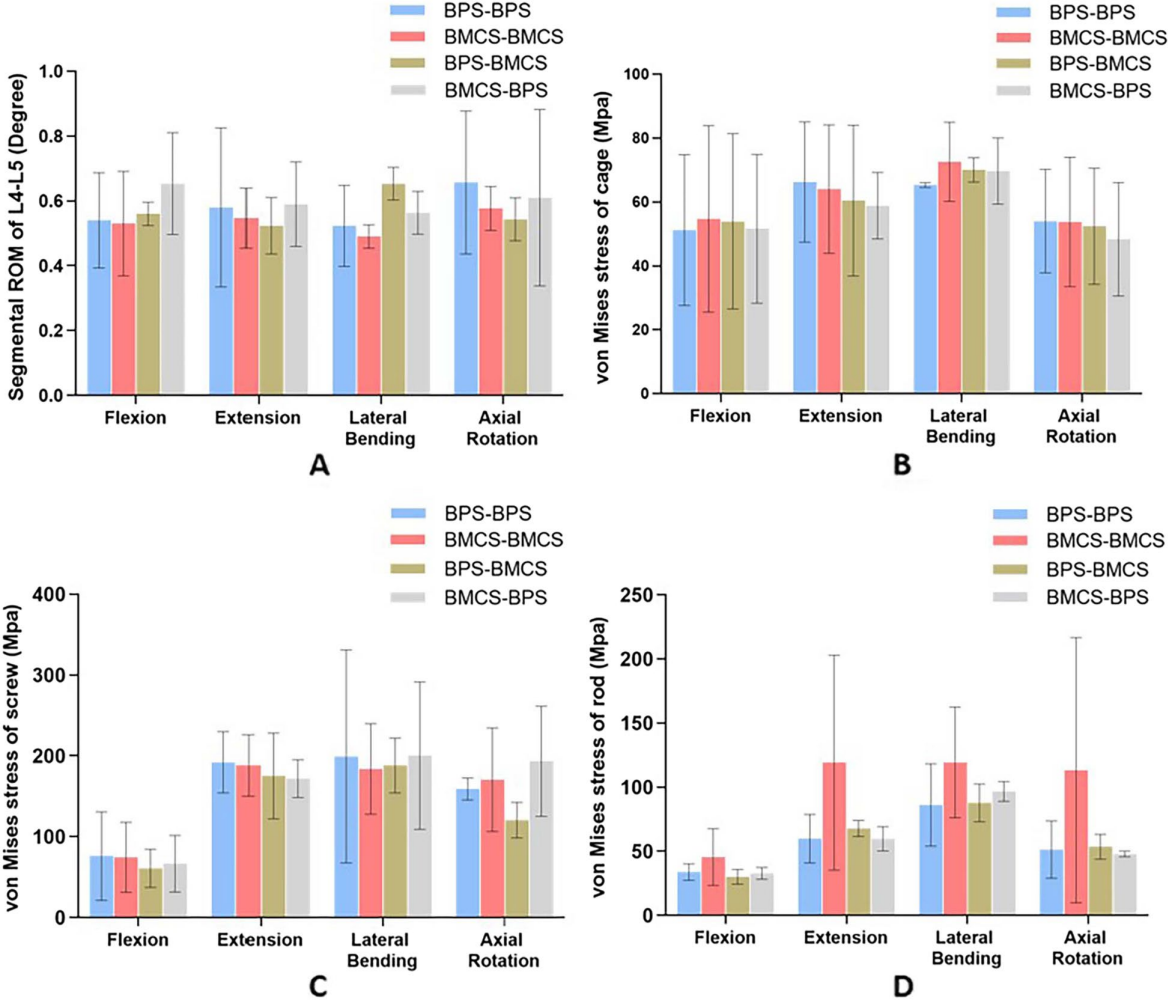
Different biomechanical results of four fixation models. **A** ROM of L4-L5 segment. **B** von Mises stress of the intervertebral cage stress at L4-L5 segment, **C** screw, and **D** rod

Compared to the BPS-BPS group, the BMCS-BMCS group showed the 1.9%, 5.7%, 5.8%, and 12.2% lower ROM in four motions. The BPS-BMCS group showed the 3.7%, and 24.8% higher ROM in flexion and lateral bending, compared to the BPS-BPS group, and has the 5.7%, and 33.3% higher ROM, compared to the BMCS-BMCS group. BPS-BMCS was decreased by 9.8%, 17.3%, 4.3%, and 5.8% in extension and rotation, respectively, compared to the BMCS-BMCS group. The BMCS-BPS group increased by 21%, 1.7%, and 7.6% in flexion, extension, and lateral bending, and decreased by 7.1% in rotation compared to the BPS-BPS group. The BPS-BMCS showed the 23.3%, 7.9%, 15%, and 5.8% increases than the BMCS-BMCS group in four motions, and the 16.7%, 12.7%, and 12.3% increases than the BPS-BMCS group in flexion, extension, and rotation, respectively, and 13.8% decrease in lateral bending (Fig. 7 A). The BPS-BMCS group showed superior stability in extension and the BMCS-BMCS group in flexion and lateral bending. Only the difference between the BPS-BMCS and BMCS-BMCS in lateral bending was significant (*P* < 0.05).

### Von Mises stress of the intervertebral cage

Compared to the BPS-BPS group, BMCS-BMCS group showed the 6.9% and 11.1% higher cage stress in flexion and lateral bending, and the 3.3% and 0.5% lower cage stress in extension and rotation. The BPS-BMCS group showed the 5.4% and 7.2% higher cage stress in flexion and lateral bending than the BPS-BPS group, and the 8.7% and 2.9% lower cage stress in extension and rotation. Compared to the BMCS-BMCS group, the BPS-BMCS group showed the 1.4%, 5.6%, 3.5% and 2.6% lower cage stress in all conditions. The BMCS-BPS group showed the same variation trend with the BPS-BMCS group, with the increase of 0.9% and 6.6% in flexion and lateral bending, and a decrease of 11.1% and 10.5% in extension and rotation, compared to the BPS-BPS group. Compared to the BMCS-BMCS group, the decrease were 5.6%, 8.1%, 4.0%, and 10.1% in four motions. Compared to the BPS-BMCS group, the reductions were 4.2%, 2.7%, 0.5%, and 7.8% in four motions (Fig. 7 B). The difference between each group was not significant (*P* > 0.05).

### Von Mises stress of the screw

**Fig. 8:**
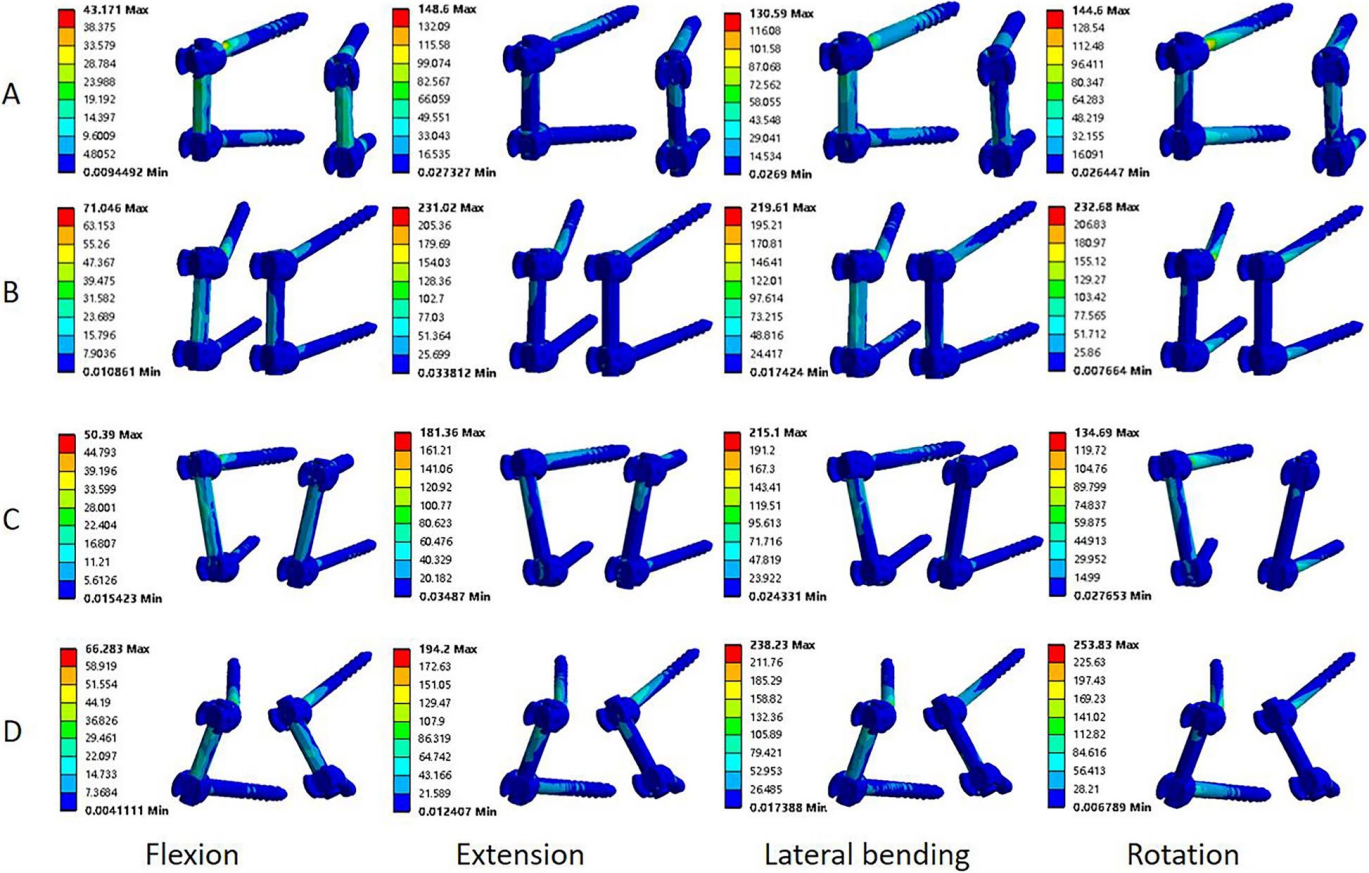
Stress nephograms over the screw in four different fixation models. **A** the BPS-BPS group. **B** the BMCS-BMCS group. **C** the BPS-BMCS group, and **D** the BMCS-BPS group

Compared to the BPS-BPS group, the BMCS-BMCS group showed the 2.0%, 2.0%, and 5.6% decrease in screw stress in flexion, extension, and lateral bending, respectively, and 7.2% increase in rotation. The BPS-BMCS group showed the 21%, 8.7%, 5.6%, and 24.3% decrease in four motions, respectively. Compared to the BMCS-BMCS group, the BPS-BMCS group showed the 18.5%, 6.8%, and 29.4% decrease in flexion, extension, and rotation, and 2.4% increases in lateral bending. Compared to the BPS-BMCS group, the BMCS-BMCS group showed the 12.7% and 10.5% decrease in screw stress in flexion and extension and the 0.5% and 21.5% increase in lateral bending and rotation. This variation trend was similar to that of the BMCS-BMCS group, where the BMCS-BPS group decreased by 11% and 8.6% in flexion–extension, and increased by 9.0% and 13.4% in lateral bending and rotation. Compared to the BPS-BMCS group, there were the increase of 9.2%, 6.5%, and 60.6% in flexion, lateral bending, and rotation, while the decrease of 1.9% in extension (Fig. 7 C). The BPS-BMCS group was superior than its counterparts in terms of screw stress, but the difference between each group was not significant (*P* > 0.05). As shown in Fig. 8, peak von Mises stress of the screw was concentrated at the screw tail connecting with the screw hub.

### Von Mises stress of the rod

**Fig. 9:**
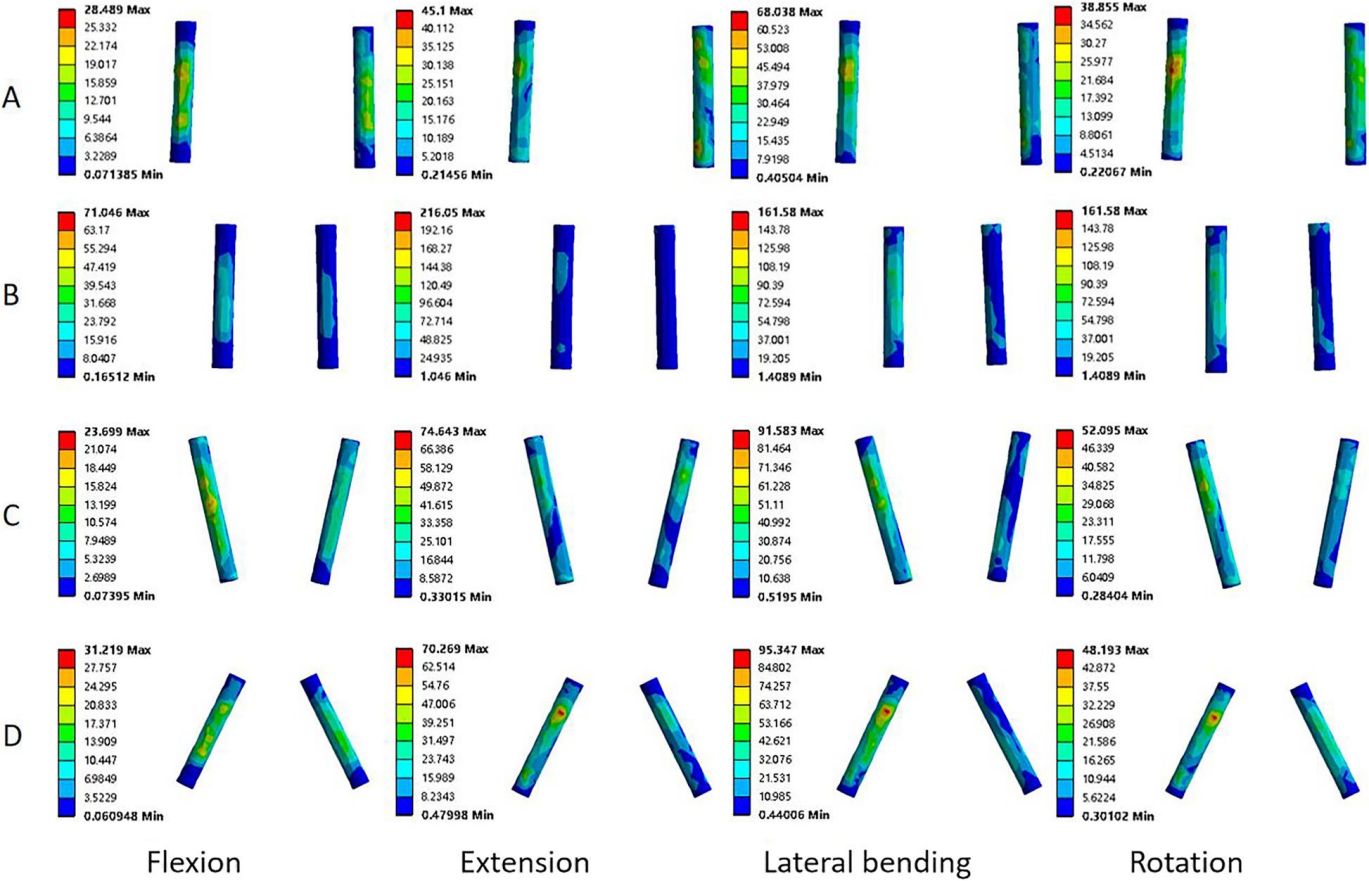
Stress nephograms over the rod in four different fixation models. **A** the BPS-BPS group. **B** the BMCS-BMCS group. **C** the BPS-BMCS group. and **D** the BMCS-BPS group

The rod stress was shown in Fig. 9. Compared to the BPS-BPS group, the rod stress of the BMCS-BMCS group increased by 34.4%, 99%, 38.6%, and 120% in four motions, respectively, and the BPS-BMCS group decreased by 11% in flexion and increased by 13.25%, 1.9%, and 4.4% in the extension, lateral bending, and rotation. Compared to the BMCS-BMCS group, the BPS-BMCS group showed the decrease of 34%, 43%, 26.5%, and 52.7% in all conditions. The BMCS-BPS group showed the decreases of 3.1%, 0.3%, and 6.6% in flexion, extension, and rotation, and the increase of 12.3% in lateral bending compared to the BPS-BPS group (Fig. 7 D). The BMCS-BPS group was superior in extension and rotation, and the BPS-BMCS group was superior in flexion and lateral bending, but the difference between each group was not significant (*P* > 0.05).

## Discussion

The superior stability of the hybrid fixation technique with MCBT and TT at the L4-L5 segment without fusion was previously demonstrated by our team [10]. However, patients with lumbar spinal stenosis were often required thorough decompression and fusion, thus the L1-S1 lumbar spine models were established and TLIF procedures were performed at the L4-L5 segment in this study. The selection of the fixation type is correlated with the occurrence of postoperative complications such as screw loosening and breakage, especially in patients with osteoporosis. As known, the CBT technique increased the pullout load by 30%, and torque by 1.7 times [6]. As previously shown by our team that the MCBT technique provides superior biomechanical stability compared to the TT and the CBT technique [8, 9]. Fujiwara et al. [26] demonstrated that increasing the medio-lateral angle and decreasing the distance to the anterior lateral edge of the upper endplate increases the screw purcahse, and further validated the rationality of our previously proposed MCBT technique. MCBT technique forms a “tri-cortical” fixation through the lamina—medial wall of the pedicle—lateral margin of the superior endplate by moving the insertion point medially, increasing the medio-lateral angle, and decreasing the cranio-caudal angle compared to the CBT technique [7–9], whereas the CBT technique only uses the cortical bone of the lamina and partial medial wall of the pedicle. However, we also found that the MCBT technique still has deficiencies, such as limited decompression of the lateral recess and intervertebral foramen, possibility of splitting the screw insertional point during decompression, and violating the thecal sac. In addition, pars fracture is the contraindication of MCBT technique. As previously shown by our team, the use of 3-dimensional printed template improves the accuracy of the MCBT screw placement [27]. If the BMCS-BMCS was implanted first, the complete decompression of the lateral recess would not be achieved. While the decompression proceeded first the screw insertion point might be damaged. It is advisable to use the hybrid BMCS-BPS and BPS-BMCS fixation techniques according to the patient’s preoperative radiographic data.

In this study, the stability of the BMCS-BMCS group in flexion and lateral bending, and the BPS-BMCS group in extension and rotation were superior to that of the BPS-BPS and BMCS-BPS groups. Perez-Orribo et al. [28] demonstrated that the stability of the CBT technique was superior in flexion and extension, but inferior in lateral bending and rotation. The differences between this study and the currenct study may be caused by the greater MCBT screw diameter of 5.0 mm and length of 40 mm compared to the CBT screw with a diameter of 4.5 mm and length ranging from 25 to 35 mm in Perez-Orribo et al. [28] and contact area between the MCBT screw and the cortical bone of the lateral edge of the upper endplate and medial wall of the pedicle. BPS-BMCS group offered the superior stability in extension and rotation, but in flexion and lateral bending. Huang et al. [29] demonstrated that the inter-screw angle of 15° between the screw at the cranial and caudal segment resulted in the lowest L4-L5 segmental ROM in flexion, extension, and rotation. In this study, the inter-screw angle was 8° in the BPS-BMCS group and 33° in the BMCS-BPS group. The morphology of the pedicle of L5 is unique in that the superior edge of the vertebral body is almost at the same level as the superior edge of the vertebral arch, but the distance between the superior edge of the pedicle of L4 and the vertebral body is somehow different from that of the L5 vertebral body, which has an “/\” pattern and moves more obliquely to the left and right, making the cranio-caudal angle of the screw in the sagittal plane more constant and milder than that of the L4 vertebral body. Therefore, when using the MCBT technique in the L5 vertebral body, the cranio-caudel angle should be reduced (around 25°) to keep the screw from penetrating the upper endplate of the cranial vertebral body, while the L4 vertebral body has a larger cranio-caudel angle (22°- 35°) [7]. Although the inter-screw angle at a sagittal plane in the BPS-BMCS group was different from that of Huang et al. [29], the variation trend of ROM in extension and rotation was similar. Except for the inter-screw angle at the sagittal plane, which in the axial plane was also different.

The peak von Mises stress of the cage was lower in the BPS-BMCS group and BMCS-BMCS group than that of in the BPS-BPS and BMCS-BMCS group in extension and rotation. In flexion and lateral bending, the cage stress of the BMCS-BMCS group showed maximum. The greater the cage stress, the higher the subsidence rate [30]. Many scholars have concluded that different types of fixation were essential to maintain the stability of the surgical segment and to reduce the cage subsidence [31], but there is still no consensus regarding the ideal fixation type. Different fixation techniques have been reported previously, but the differences among the BPS-BPS, BMCS-BMCS, BPS-BMCS, and BMCS-BPS techniques have not been reported. The previous study showed that the BMCS-BPS group had the lowest intervertebral disc stress in rotation. The BMCS-BPS group in the current study showed the lowest cage stress (51.7 ± 23.3 MPa) in rotation, which was comparable to the variation trend in the previous model without fusion[10]. In the biomechanical analysis of Xu et al. [31], the peak von Mises stress of cage was found in lateral bending, and the peak von mises stress of the single-cage (77.23 MPa) was greater than paired-cage (49.77 MPa) in TLIF model with BPS-BPS technique [32]. In this study, the peak von Mises stress of cage was found in lateral bending in the BMCS-BMCS group (72.66 ± 12.4 MPa), but in the BPS-BPS group. The peak von Mises stress of cage was found in extension (66.27 ± 18.85 MPa), variation trend of BPS-BPS group was inconsistent with Xu et al. [31]. This difference may be caused by the different screw diameters, lengths, and cage sizes. The mean cage stress in the BMCS-BPS group was lowest in extension and rotation and lower in flexion and lateral bending, but the difference was not statistically different (*p* > 0.05). In the randomized control trial of Lee et al. [32], the fusion rates of CBT and pedicle screw technique were comparable (CBT: 94.5%, PS: 91.4%, *P* > 0.05) at 24 months postoperatively, but the MCBT technique has not been used in the clinical practice yet. There was no statistical difference in the stability between the single and paired cage in the TLIF model [12]. Single cage insertion was preferable because of its convenience, and lower cost. As for the hybrid fixation with the MCBT and TT, the different effects of single and paired cages on the stability of the fixation and fusion rate need further biomechanical study.

Increasing the medio-lateral angle of the pedicle screw reduced the risk of screw loosening and breakage [33]. Peak von mises stress of the screw in the hybrid fixation technique with CBT and pedicle screw, in Su et al. [34], was lower than that of in BPS-BPS technique in flexion, extension, and lateral bending. In this study, As for the BPS-BMCS group, the von Mises stress of the screw was lower than those of the BPS-BPS group in all motions. As for the BMCS-BPS group, the von Mises stress of the screw was lower than that in the BPS-BPS group in flexion, extension, and rotation, the stress variation trend was comparable to the Su et al. [34]. BPS-BMCS group and BMCS-BPS group may reduce the incidence of screw breakage. The lowest screw stress was found in the BPS-BMCS (30.12 ± 5.73 MPa) and BMCS-BPS (32.79 ± 4.63 MPa) group in flexion. As for CBT-CBT (CBT at L3 and L4), the von mises stress of the screw was greater than that of the counterparts in flexion and rotation [34]. However, the opposite result was found in the current study, which may be related to the greater medio-lateral angle of the MCBT technique than that of the CBT technique as mentioned by Newcomb et al. [33]. Huang et al. [29] suggested that the longer rod length may decrease the screw stress. In this study, the BMCS-BPS group had the shortest rod length and the highest screw stress in lateral bending (200.34 ± 91.11 MPa) and rotation (193.35 ± 68.09 MPa), while the BPS-BMCS group had the longest rod length and the lowest von mises stress of the screw in flexion (60.81 ± 23.70 MPa) and rotation (120.37 ± 21.98 MPa) (*P* > 0.05). Results in rotation was consistent with Newcomb et al. [33], the reasons were probably due to the different sample sizes, different material properties, and inconsistent screw and rod sizes in the two studies. As shown in Fig. 8, the maximum stress in the screw occured near to the screw hub, that is, the BPS-BMCS group may reduce the risk of screw breakage.

Commonly, the junction of the screw and the connecting rod is the most common site for breakage. In this study, the von Mises stress of the rod in the BMCS-BMCS group was greater in all motions than its counterparts, with the maximum stress occurring in lateral bending (119.35 ± 43.20 MPa) at the lower end of the junction between the screw and the connecting rod (Fig. 8). The rod stress of the BMCS-BPS group was lower than that of the counterparts in flexion (32.79 ± 4.63 MPa), extension (59.72 ± 9.46 MPa), and rotation (47.92 ± 2.29 MPa), and the BPS-BMCS group (86.15 ± 32.15 MPa) showed the rod stress approximately similar to that of BPS-BPS group (87.76 ± 14.74 MPa) in lateral bending. Rod breakage and screw breakages are related to the high stress level of the rod and screw [35]. The different screw insertion points and screw trajectories of the MCBT and TT techniques in this study resulted in different rod stress with the different combinations of fixation techniques. It can be deduced that the BPS-BMCS and BMCS-BPS groups may reduce the risk of rod breakage. Wang et al. [36] showed that the CBT-CBT group (CBT screw: 5.0 mm in diameter, 35 mm in length) had the highest rod stress in almost all conditions. Although the MCBT screw (5.0 mm in diameter, 40 mm in length), in this study, had the different trajectory and slightly longer length than the CBT screw in the study of Wang et al. [36], the variation rule of the rod stress was consistent. Xiao et al. [37] demonstrated that the Dynesys hybrid fixation technique combined the advantages of dynamic stabilization and rigid fusion, using a “soft” rod in the upper segment to reduce the incidence of adjacent segment degeneration (ASD), further manifested the principle of “overcoming rigidity with flexibility”. By analogy, the effect on the adjacent segment of the “rigid” fixed segment using the hybrid fixation techniques and connecting with the “soft rod” needs to be further investigated.

Lumbar musculature plays an important role in spinal stability [38]. A previous study showed that S1 screw loosening was closely related to the degeneration of paraspinal muscles [39]. CBT screw reduces the postoperative serum creatinine phosphokinase concentration [40]. Since the insertion point of the MCBT technique is closer to the midline than the CBT technique [7, 8], the hybrid technique can further reduce the damage to the facet joint [10] and iatrogenic paravertebral muscle injury. Compared with the traditional BPS-BPS and CBT-CBT fixation techniques, the hybrid BPS-BMCS and BMCS-BPS techniques can not only overcome the embarrassing situation of incomplete decompression of the lateral recess after implantation of the screw but also reduce the surgical incision to reflect the principle of “minimally invasive surgery” and avoid the degeneration of the adjacent segment.

Perez-Orribo et al. [28] demonstrated that CBT offered greater rigidity in flexion and extension. However, it is worth noting that 20% of CBT screws may cause medial pedicle wall fractures resulting in nerve injury [6]. The medio-lateral angle of the MCBT technique is greater than that of the CBT technique, and it is extraordinarily difficult to complete the high precision screw placement with freehand in high accuracy, therefore a 3-dimensional printed template or a robotic technique is required to assist the screw placement. Our research team has previously investigated the efficacy of 3-dimensional printed template in 3-dimensional printed vertebrae and human cadaveric lumbar wet specimnes using MCBT technique, and demonstrated that it can improve the accuracy of the MCBT screw placement [27].

There were some limitations in this study. First, the muscle tissues were not reconstructed, which may affect the stability of the lumbar spine. Second, this study did not analyze the effect of the hybrid technique on the adjacent segments in the TLIF model. Third, screw sizes of different diameters and lengths were not discussed in this study. Fourth, the sample size of this study is only three which needs to be increased in further study.

## Conclusion

This study used finite element analysis to evaluate the biomechanical effects of four different fixation techniques in the L1-S1 lumbar spine with the TLIF model at the L4-L5 segment. The BMCS-BMCS and the BPS-BMCS technique provide superior stability than the BPS-BPS and BMCS-BPS technique. BPS-BMCS technique presented a lower risk of screw breakage and BMCS-BPS technique presented a lower risk of rod breakage. The BPS-BMCS and BMCS-BPS techniques may reduce the cage subsidence rate. The results of this study support that the use of the BPS-BMCS and BMCS-BPS techniques in TLIF for offering superior stability and a lower instrument-related complication. Although the BPS-BMCS and BMCS-BPS may reduces the risk of cage subsidence, the effect of different cage types to the cage subsidence need to be further investigated.

## Data Availability

The raw data supporting the conclusion of this article will be made available by the Corresponding author, without undue reservation.

## Abbreviations

TLIF: Transforaminal lumbar interbody fusion
PLIF: Posterior lumbar interbody fusion
TT: Traditional trajectory
CBT: Cortical bone trajectory
BPS: Bilateral pedicle screw
BMCS: Bilateral modified cortical bone trajectory screw
FE: Finite element
ROM: Range of motion
ALL: Anterior longitudinal ligament
PLL: Posterior longitudinal ligament
ITL: Intertransverse ligament
LF: Ligamentum flavum
CL: Capsular ligament
ISL: Interspinous ligament
SSL: Supraspinous ligament
BMD: Bone mineral density
